# On the Inspiration of Vapor in Certain Lesions of the Breathing Apparatus

**Published:** 1865-01

**Authors:** John Hunter


### ON THE INSPIRATION OF VAPOR IN CERTAIN LESIONS OF THE BREATHING APPARATUS.

By JOHN HUNTER, M. A.

Without laying claim to any great originality, I trust that the few observations I have to make upon this important subject, to which I have directed considerable attention, may be worthy of notice by the profession.

I suppose no one will deny that, as a general principle, the use of moisture in combination with air is of service in chest diseases. This we see daily evidenced by the beneficial effect of sea-air—which contains vapor in excess of the amount held by the air generally—in different forms of bronchitis, etc. But it is to insist on the powerful remedial agent which we possess in vapor when judiciously used at the bedside of the patient that is the object of the present paper. On the first appearance of an attack of, say, simple bronchitis, the first system observed by the patient is a feeling of oppression in breathing, "a tight feeling across the chest." This is caused by the proper secretion of the lining membrane of the lungs being partially arrested. The air-cells are in an equal degree incapable of performing their proper functions—namely, the reception of oxygen from the inspired air, and the giving out of carbonic acid gas; the result of which is that the patient has a diminished area of breathing-surface, in proportion to the intensity of the attack. And here I may remark, in passing, that there is an evident necessity in such a case that the patient should have the purest air procurable, to make some amends for the deficient quantity. Now in this stage of the bronchitis, I believe we have no such powerful agent as steam, properly applied. The precise mode of application may be varied to suit different attacks in patients of different ages, and other peculiarities; the great principle being that the steam should be carried with the inspired air into the air-cells, where it not only acts as a substitute for the natural mucous secretions, but by its soothing action disposes the membrane to resume its normal functions. I have seen an attack of acute bronchitis cut short by these means, almost without any other; while in the different forms of asthma it is found of the greatest service. I put the acute bronchitis first in the list, as I think that it yields to the power of steam more easily than any other form of chest-disease; but it is good in all. And even in chronic bronchitis it is of the greatest service in softening the glutinous sputa, and rendering it much more easy to be got up by the patient, thereby saving time and many coughs, the patient being enabled to get up all his expectoration at the beginning of the night, and thus start, as it were, with a fresh account, instead of being tormented by the small installments frequently due. In phthisis, also, I have found the breathing-in of steam to be of the greatest service in the frequent though often slight attacks of localized congestion, which denote that the disease is placing its grasp upon another portion of the lung; such attacks, if not altogether warded off, being certainly modified in their intensity. I could now give details of many cases in which phthisis appeared to be kept in subjection, each exacerbation of the disease being speedily met and apparently crushed; but I must proceed to give details of the mode of application of the steam. This varies in different patients.

In the case of infants, where the simplest means must be used, I have found nothing to succeed so well as placing a flat and broad vessel at each side of the cot in which the little patient is; these should be kept filled by the nurse with hot water, and the steam from them is caused to ascend and float round the little sufferer, who unconsciously inspires the beneficial vapor. In patients of more mature age the steam may be applied by an ordinary teapot, which being *half* filled with boiling water, and the mouth applied to the spout, the patient may be taught to draw in the steam with his inspirations. At first it may give rise to a little irritation of the mucous membrane, but the patient quickly feels the beneficial effect, and generally perseveres in its use. It is a good plan to put some tea, cinnamon, etc., at the bottom of the teapot, which prevents the insipid taste of the steam from being disagreeable to the patient. I lately had a patient, an overlooker in a cotton factory, who was suffering from chronic irritation of the lungs, evidently arising from the small particles of cotton floating with the inspired air into his air-cells, and there giving rise to his sufferings. I explained to him the action of steam in his case, and he, being of an ingenious turn, put a piece of elastic tubing, about four feet long, to the spout of the kettle, and after his day's work, regularly sat by his fire inhaling the steam, and in about half an hour was able to get up all the cotton taken in during the day, thereby ensuring a comfortable night's rest, which had been before impossible from cough. Steam may be applied in this way: a small perforation is made in the lid of a common kettle, into which may be soldered a short tin tube bent up at a right angle; attached to this may be a tube of vulcanized india-rubber, of any reasonable length, which may reach over to the bed-side of the patient, and, opening out near the patient's mouth, may during the night keep the air inspired hot and moist. The whole expense of this apparatus is only a few shillings. This plan is adapted for night-work, as the kettle, if filled at bedtime, will furnish steam sufficiently during the whole night. Many other plans may be adopted, and no doubt will occur to the ready mind, but the great object to be attained is to keep the air inspired warm and moist as a powerful curative agent, being one particularly useful in this climate, where diseases of the chest are so common.—*London Lancet.*

---

Laudanum and Teetotalism.—Dr. Alfred Taylor, commissioned by the Privy Council, has sent in a report on the means of committing murder by poisons which are allowed to exist in England. He says that poison enough to kill two adults can be purchased anywhere for threepence; and that the careless dispensing of poisonous drugs is cause of most frightful accidents. As to laudanum, it appears to be sold wholesale, single shops in the Marshland supplying three or four hundred customers every Saturday night. Retail druggists often dispense 200 lbs. in one year, and one man complained that his wife had consumed £100 in opium since he married. It is a mistake to consider the practice confined to the marshy districts. We do not believe there is a town in England where some one chemist does not on Saturday night load his counter with little bottles of laudanum; and we are assured by a wholesale druggist that he could and did sell it in the eastern counties to the extent of some thousands of pounds in a year. This gentleman, an old and keen observer, declared that the demand had sprung up shortly after the introduction of teetotalism, and that it would be found to vary everywhere, in accordance with the progress or decline of the system of total abstinence.—*London Spectator.*

